# Virulence and antimicrobial-resistance of shiga toxin-producing *E. coli* (STEC) Isolated from edible shellfish and its public health significance

**DOI:** 10.1007/s00203-022-03114-2

**Published:** 2022-07-21

**Authors:** Dheyazan M. Ali Al Qabili, Abdel-Karim M. Aboueisha, Ghada A. Ibrahim, Ahmed I. Youssef, Heba S. El-Mahallawy

**Affiliations:** 1 Department of Animal Hygiene, Zoonoses, and Animal Behaviour and Management, Faculty of Veterinary Medicine, Suez Canal University, Ismailia, 41522 Egypt; 2 Bacteriology Department, AHRI, Ismailia branch, Ismailia, 41511 ARC Egypt

**Keywords:** Shellfish, *E. coli*, STEC, Antimicrobial resistance, Virulence genes, PCR

## Abstract

Shiga toxin-producing *E. coli* (STEC) are an important cause of foodborne illness in humans with infections ranging from mild non-bloody diarrhea to bloody diarrhea (BD) and hemolytic uremic syndrome (HUS). This study aimed to investigate the distribution of STEC in shellfish from coastal shores of Lake Timsah in Ismailia Governorate, Egypt and its probable hazard to seafood consumers. Samples from the external surface and tissues of shrimp (*n* = 45), crabs (*n* = 45), and oysters (*n* = 45) batches were examined bacteriologically for the presence of STEC and tested for their antibiotic sensitivity. Moreover, occurrence of virulence genes was determined via detection of *stx1*, *stx2* and *eaeA* genes using PCR. Overall, *E. coli* and presumptive STEC isolates (from CHROMagar) were identified from the surface (55.6 and 5.9%) and tissues (42.2 and 8.9%) of the examined shellfish batches, respectively. Five STEC isolates had been confirmed and found belonging to O26:H11, O125:H6, O146:H21, and O159 serogroups, those were 4 isolates from tissues of the three shellfish species and one isolate from the crab surface. The STEC isolates were multi-drug resistant, showing complete resistance to; penicillins, amoxycillin/clavulanic acid, colistin, fosfomycin, ceftriaxone, ciprofloxacin, and tetracycline, however, they were sensitive to gentamycin except O159 serogroup. The current study revealed low level of contamination of shellfish from coastal shores of Lake Timsah with STEC, however, it also highlights the extreme level of antimicrobial resistance exhibited by the presumptive and confirmed STEC isolates which is very hazardous for seafood consumers in the study area.

## Introduction

*Escherichia coli* has been long thought to be a sign of faecal contamination in water and food. There are over 700 *E. coli* serotypes, however, the majority of which are non-pathogenic and present as commensals in the human and animal gastrointestinal tract. A limited number of *E. coli* pathotypes have developed the potential to produce a wide range of illnesses in humans ranged from wound infections to deadly meningitis (Jang et al. 2017). Intestinal *E. coli* can be classified into at least five types, with the Shiga toxin-producing *E. coli* (STEC), (known also as enterohemorrhagic *E. coli,* EHEC), being the most dangerous (Prakasan et al. 2018).

Shellfish including bivalves and gastropod mollusks (cockles, oysters, mussels, clams, periwinkles, and sea-snails), as well as, crustacean shellfish (lobster, crab, and shrimp) are preferable as rich sources of protein for humans, however, their contamination with bacteria found in human feces is still considered a health risk (Costa 2013). STEC can infect individuals inhabiting marine environment through contaminated seafood and by contaminated water and food; where bacteria colonize the intestinal microvilli before attacking the central nervous system and kidney cells (Kamala et al. 2022; Sujatha et al. 2011).

STEC infections cause 100,000 illnesses, 3000 hospitalizations, and 90 fatalities per year in the United States alone (Gomes et al. 2016). The development of one or more Shiga toxins is the STEC’s key virulence factors. There are two types of Shiga toxins (*Stx*); *Stx1*, which is nearly identical to the toxins generated by *Shigella dysenteriae* 1, and *Stx2*, that is roughly 60% similar to *Stx1* (Steiner 2016). Although the formation of one or more Shiga toxins is required to produce illness, the production of *Stx2* is more closely linked to the severity of diseases like haemolytic uremic syndrome (HUS) and hemorrhagic colitis (HC) (Melton-Celsa 2014).

Additional to the synthesis of one more Shiga toxin, numerous supplementary virulence factors are known to influence STEC pathogenicity in human infections. The ability to create attachment and effacement lesions (A/E lesions) is one of the significant virulence features of STEC (Smith et al. 2014). Although the exact role of these virulence factors is unknown (Kamala et al. 2022), these virulence factors have been found in STEC reported from clinical cases of HC and HUS on a regular basis, if not always. Despite O157:H7 is the most prevalent serotype that has been linked to the majority of large-scale STEC outbreak infections, non-O157 serotypes such O26, O91, O45, O111, O103, O145, and O121 are also involved in serious human infections (Luna-Gierke et al. 2014).

Lake Timsah is one of the important coastal areas in Ismailia Governorate, Egypt, with a salinity ranges from 20–40% in surface areas and more than 40% in deeper ones (El-Serehy et al. 2018). Although ruminants are the main reservoirs for STEC, other sources including wildlife, marine environment, and aquaculture could be possible spillover hosts for these bacteria (Kamala et al. 2022; Kim et al. 2020). Although previous studies have documented presence of several pathogens, there have been few reports on STEC spreading densities from coastal and marine environment (Baliere et al. 2015; Kamala et al. 2022; Martin et al. 2019; Prakasan et al. 2018). The present study aims to investigate to which level STEC and their virulence factors are present on the surfaces and accumulated in the tissues of shellfish at harvesting areas of Lake Timsah, Ismailia, Egypt.

## Material and methods

### Ethical statement

Sample collection and laboratory procedures for the present study was reviewed and approved by the Ethical Committee of Faculty of Veterinary Medicine, Suez Canal University, Egypt (No. 2017003).

### Sample collection and preparation

A total of 135 pooled fresh marine shellfish batches (45 shrimp, 45 crabs, and 45 oysters) were collected from different locations along the Shore of Lake Timsah at Ismailia governorate. Collection locations were the common harvesting sites where local fishermen collect the shellfish to be marketed for consumers. Samples from each collection batch were kept separately in a plastic bag and transferred in an ice box within 1 hour of collection to the Zoonoses Department’s Laboratory, Faculty of Veterinary Medicine, Suez Canal University for processing and isolation of *E. coli*.

### Isolation and identification of *E. coli* and STEC detection

Soft tissue samples; about 25 g of each shellfish species batch comprised of 7–15 individual shrimp, crab or oyster, were pooled to represent one sample, placed in a sterile stomacher bag with 225 ml *E. coli* broth (Biolife, Italiana) and homogenized in stomacher (Lab. Blender 400, Seward Lab, London) for 3 min. 1 mL of the homogenized tissue was incubated with 9 mL of *E. coli* broth at 37 ℃ for 24 h (Doyle et al. 2020). Moreover, sterile swabs were rolled over the outer surface of the pooled shellfish samples, immersed in *E. coli* broth (Biolife, Italiana) and incubated at 37 ℃ for 24 h. Loopfuls from each surface swab and tissue’s *E. coli* broth culture were sub-cultured on MacConkey agar (Himedia, India) and EMB agar (Himedia, India) that were incubated at 37 ℃ for 24 h. Typical *E. coli* colonies on EMB (greenish metallic sheen colonies in reflected light) were re-cultured until pure colonies were obtained (Doyle et al. 2020). Isolates were biochemically identified using IMVIC tests and isolation on TSI as previously described (Thampuran et al. 2005). For selective STEC detection, pure identified *E. coli* colonies were grown on CHROMagar STEC agar medium (CHROMagar Microbiology, Paris, France) and incubated at 37 ℃ for 24 h (Meng et al. 2012).

### Serological identification of presumptive STEC isolates

Presumptive STEC isolates successfully grown on CHROMagar agar medium (mauve colonies) were serologically identified in the Food analysis Center at Faculty of Veterinary Medicine, Benha University. Rapid diagnostic *E. coli* polyvalent antisera sets against O and H antigen for identification of the Enteropathogenic types were used as previously described (Kok et al. 1996).

### Virulence profile of presumptive STEC isolates from shellfish

Virulence of presumptive STEC isolates were assessed using PCR for 3 virulence genes *stx1*, *stx2*, and *eaeA* (Table 1). The QIAamp DNA Mini kit (Qiagen, Germany, GmbH) was used to extract bacterial DNA based on the manufacturer’s instructions. Duplex PCR reactions for *stx1* and *stx2* was performed in a 50 µl reaction mixture, containing 25 µl of EmeraldAmp Mix PCR Master Mix (Takara, Japan), 1 µl of each primer (20 pmol concentration) (Metabion, Germany), 15 µl of molecular grade water, and 6 µl of bacterial DNA. Moreover, a uniplex PCR reactions for *eaeA* attachment gene was performed in a 25 µl reaction mixture, containing 12.5 µl of EmeraldAmp Mix PCR Master Mix (Takara, Japan), 1 µl of each primer (20 pmol concentration) (Metabion, Germany), 5.5 µl of molecular grade water, and 5 µl of bacterial DNA. The PCR reactions were performed in an Applied biosystem 2720 thermal cycler. PCR products (20 µl) were separated by electrophoresis at room temperature against 100 bp ladder (Fermentas, Germany) through 1.5% agarose gel (Applichem, Germany, GmbH) in 1 × TBE buffer. The gel was then photographed using Gel Documentation System (Alpha Innotech, Biometra).

**Table 1:** Primers sequences, cycling conditions, and expected product size of virulence genes in presumptive STEC isolates

Target gene	Primers sequences	Product size (bp)	Prim. Den	Amplification (35 cycles)			Final extension	References
				Den	Ann	Ext		
*stx1*	ACACTGGATGATCTCAGTGG CTGAATCCCCCTCCATTATG	614	94 ℃ 5 min	94 ℃ 30 s	58℃ 40 s	72 ℃ 45 s	72 ℃ 10 min	(Dipineto et al. 2006)
*stx2*	CCATGACAACGGACAGCAGTT CCTGTCAACTGAGCAGCACTTTG	779						
*eaeA*	ATG CTT AGT GCT GGT TTA GG GCC TTC ATC ATT TCG CTT TC	248	94 ℃ 5 min	94 ℃ 30 s	51 ℃ 30 s	72 ℃ 30 s	72 ℃ 7 min	(Bisi-Johnson et al. 2011)

### Antimicrobial sensitivity of presumptive STEC isolates from shellfish

Identified STEC isolates were tested for their sensitivity against 12 antibiotics from different antimicrobial classes using disc diffusion method on Muller Hinton agar (Oxoid, UK). Antibiotic discs (Oxoid, UK) with following concentrations were used: ampicillin (AM) 10 µg, piperacillin (PRL) 100 µg, amoxycillin/clavulanic acid (AMC) 30 µg, ceftriaxone (CTR) 30 µg, azithromycin (AT) 15 µg, chloramphenicol (C) 30 µg, ciprofloxacin (CIP) 5 µg, colistin (CT) 10 µg, fosfomycin (FF) 50 µg, gentamycin (Gen) 10 µg, trimethoprim sulphamethoxazole (SXT) 25 µg, and tetracycline (TE) 30 µg. Pure freshly grown STEC isolates were diluted in 5 ml Muller Hinton broth (Oxoid, UK) to a density = 0.5 MacFarlane standard. Sterile swabs were used to evenly streak the surface of MHA plates and left for 30 min. to dry. Using sterile forceps, antibiotic discs were placed firmly to the plate and incubated at 37℃ for 18 h before measuring the inhibition zone as recommended by the manufacturer and Clinical and Laboratory Standard Institute (CLSI 2020), where isolate were categorized as resistant, intermediate or sensitive.

## Results

Out of the examined shellfish batches from (45 oyster, 45 crabs, and 45 shrimp), the surface had higher *E. coli* and presumptive STEC detection rate (55.6 and 5.93%) than the edible tissues (42.2 and 8.89%). Among the total *E. coli* isolates, 20 presumptive STEC isolates showed characteristic mauve colonies on CHROMagar STEC medium; 12 isolates from tissues (8.89%) and 8 isolates from the external surface (5.93%) of the examined shellfish specimens. The recovery rate of *E. coli* and presumptive STEC from surface and tissues among different shellfish species (shrimp, crabs, and oysters) was nearly similar, except the tissues from oysters had the highest occurrence (11.11%) (Table 2).

**Table 2:** Total *E. coli* and virulence genes of presumptive STEC isolated from tissues and surface of shellfish samples

Type of samples	Total No. of samples		Positive *E. coli*	Presumptive STEC from CHROMagar in relation to the total examined samples	Serotype of presumptive STEC isolates	Virulence genes in presumptive STEC isolates		
			No. (%)	No. (%)		*eae*A	*stx1*	*stx2*
Tissue	Oyster	45	18 (40.00)	5 (11.11)	O44:H18	−	−	−
					O44:H18	+	−	−
					O125:H6	−	−	−
					**O125:H6**	−	+	−
					O128:H2	−	−	−
	Crab	45	21 (46.67)	3 (6.67)	O128:H2	−	−	−
					**O159**	+	−	+
					Untypable	+	−	−
	Shrimp	45	18 (40.00)	4 (8.89)	O117:H4	+	−	−
					O117:H4	+	−	−
					**O26:H11**	−	−	+
					**O146:H21**	+	−	+
Total		135	**57 (42.2)**	**12 (8.89)**		**6**	**1**	**3**
Surface	Oyster	45	24 (53.33)	0 (0.00)	ND	ND	ND	ND
	Crab	45	27 (60.00)	4 (8.89)	O55:H7	−	−	–
					O55:H7	−	−	–
					**O146:H21**	+	+	–
					O128:H2	−	−	−
	Shrimp	45	24 (53.33)	4 (8.89)	O117:H4	+	−	−
					O117:H4	+	−	−
					O44:H18	+	−	−
					O103:H2	+	−	−
Total		135	**75 (55.60)**	**8 (5.93)**		**5**	**1**	**−**

ND: not determined

Recovered presumptive STEC isolates belonged to 9 serotypes (O26:H11, O55:H7, O103:H2, O44:H18, O117:H4, O125:H6, O128:H2, O146:H21, and O159) with O117:H4, O128:H2, O44:H18 were the most frequent serotypes (Table 2). Molecularly, 11 presumptive STEC isolates carried *eaeA* gene (55%), 3 isolates carried s*tx2* gene (15%) and only 2 isolates had s*tx1* (10%) virulence gene. Of these, three isolates carried *eaeA* mutually with either *stx1* or *stx2* (Table 2). Confirmed STEC isolates (5 isolates, 4 from tissues of oyster, crabs, and shrimp, and one from crab surface that were positive for *stx1* or *stx2*) belonged to O26:H11, O125:H6, O146:H21, and O159 serogroups.

**Fig. 1:**
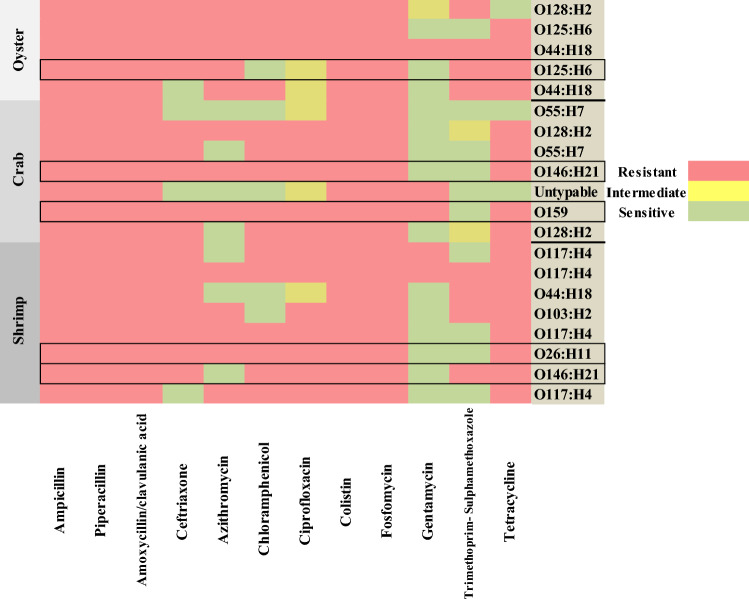
Heat map showing antibiogram of presumptive STEC serotypes from oyster, crabs and shrimp. The highlighted rows represent the confirmed STEC serotypes that were positive for *stx1* or *stx2*

The overall antimicrobial sensitivity test results for presumptive STEC isolates is shown in (Table 3 and Fig. 1). The confirmed STEC isolates were multi-drug resistant (MDR), where isolates showed complete resistance to; ampicillin, piperacillin, amoxycillin/clavulanic acid, colistin, fosfomycin, ceftriaxone, ciprofloxacin, and tetracycline, however, they were sensitive to gentamycin except O159 serogroup.

**Table 3:** Antimicrobial susceptibility test results of presumptive STEC isolates recovered from surface and tissues of shellfish

Antimicrobial class	Antibiotic Agen and concentration	Presumptive STEC (*n* = 20)		
		Resistant No. (%)	Intermediate No. (%)	Sensitive No. (%)
Penicillins	Ampicillin (10 µg)	20 (100)	0 (0.0)	0 (0.0)
	Piperacillin (100 µg)	20 (100)	0 (0.0)	0 (0.0)
Β-lactams combination agents	Amoxycillin/clavulanic acid (30 µg)	20 (100)	0 (0.0)	0 (0.0)
Cephems	Ceftriaxone (30 µg)	16 (80)	0 (0.0)	4 (20)
Macrolides	Azithromycin (15 µg)	13 (65)	0 (0.0)	7 (35)
Phenicols	Chloramphenicol (30 µg)	15 (75)	0 (0.0)	5 (25)
Fluoroquinolones	Ciprofloxacin (5 µg)	15 (75)	5 (25)	0 (0.0)
Lipopeptides	Colistin (10 µg)	20 (100)	0 (0.0)	0 (0.0)
Fosfomycins	Fosfomycin (50 µg)	20 (100)	0 (0.0)	0 (0.0)
Aminogylcosides	Gentamycin (10 µg)	5 (25)	1 (5)	14 (70)
Folate pathway antagonists	Trimethoprim- Sulphamethoxazole (25 µg)	8 (40)	2 (10)	10 (50)
Tetracyclines	Tetracycline (30 µg)	17 (85)	0 (0.0)	3 (15)

*ND* not determined.

## Discussion

Shellfish can concentrate many important pathogenic bacteria of human food borne transmission from the surrounding waterways by their filter-feeding activities (Walker et al. 2020), consequently, their consumption may result in food poisoning outbreaks.

Results from the present study revealed that, shellfish samples from coastal shores of Lake Timsah harbored virulent multi-drug resistant STEC (Table 2 & 3). The present findings showed higher *E. coli* detection rate from crabs’ tissues and surface samples (46.7 and 60%, respectively) than oysters and shrimp (40 and 53.3%, in both of them). The prevalence of *E. coli* from the surface was higher than that from the tissues in the 3 studied shellfish species. This may be because the surface is more exposed to contaminated surrounding water. A total of 150 *E. coli* isolates were recovered from 4 fresh shellfish species in Mumbai, India (Singh et al. 2020). Moreover, in another study, all the examined shellfish samples from landing centers and retail fish markets were positive for *E. coli* (100%) (Prakasan et al. 2018). In this regard, Martin and his co-authors reported that any edible marine shellfish species found exposed to fecal contamination, could be regarded as a potential source of STEC (Martin et al. 2019).

The significant increase in STEC outbreaks over the past decade as a consequence to ingestion of contaminated food or contact with animals and animal products is a public health issue (Kim et al. 2020). Out of the total *E. coli* isolates in this study, 5 STEC isolates were confirmed (4 from tissues of the three shellfish species and one from crab surface). Similarly, STEC (5%) were detected in shellfish samples from coastal harvesting areas in France (Baliere et al. 2015) and a very low level of STEC (3/269, 1.1%) was reported from Norwegian bivalves (Martin et al. 2019). However, a huge number, 3742 STEC isolates, were recently reported from fish, coastal waters, and sediments of Southeast Coast of India (Kamala et al. 2022). In Europe, besides O157:H7, 4 serotypes; O26:H11, O103:H2, O145:H28, and O111:H8 are the most commonly implicated in human STEC outbreaks, comprising the five highly pathogenic serotypes (EFSA 2013). In this regard, the first 2 of these serotypes had been detected from shrimp tissues and external surface in the present study, however, the O103:H2 didn’t carry any of the shiga toxin genes and only carried the *eae*A gene (Table 2). Generally, *E. coli* organisms in fish and fishery products are very alarming for human health, as well as, for fish export earnings. Overall, the quality of fresh fish could be considered as a strong sanitary indicator for bacterial contamination (especially *E. coli*) and unhygienic conditions of water or fish aquarium (Jang et al. 2017), thus understanding food sources for STEC and possible reservoirs is very crucial.

STEC are identified as those strains that produce shiga toxins (encoded by *stx*_*1*_ and *stx*_*2*_ genes). Presence of virulence-associated genes is the primary differentiating feature between pathogenic and non-pathogenic *E. coli* strains. Using PCR, in the present study, *eaeA* was the most prevalent (55%) virulence gene in presumptive STEC isolates, however, *stx1* and *stx2* were detected in only 5 isolates; with 3 isolates (O159 and O146:H21) mutually carried *eae*A with either *stx1* or *stx2*. A higher level of *eae* (74.8%) and *stx* (30.3%) genes were detected in shellfish samples from coastal harvesting areas in France (Baliere et al. 2015). However, STEC from marine sediments and shellfish in Morocco lack the intimin *eae* gene (Bennani et al. 2011). In India, 1518 out of 3742 STEC isolates from fish, coastal waters, and sediments of Southeast Coast carried the *stx*1, *stx*2, and *eae* virulence genes (Kamala et al. 2022). Also, *stx*1, *stx*2, and *eae* virulence genes were detected at lower rate (19/269, 7%) from STEC isolates recovered from marine Norwegian bivalves (Martin et al. 2019). Presence of such virulent STEC isolates is of especial interest for shellfish consumers in the study area, as most of these edible shellfish species usually cooked by steaming and didn’t receive sufficient heat treatment during cooking.

Distribution of MDR bacteria is a global public health threat. Commercial fish could be a vehicle of antibiotic-resistant human pathogens that may be indirectly transmitted to human causing sever public health hazard (Singh et al. 2020). According to sensitivity testing results in the present study, all presumptive STEC isolates were multi-drug resistant (MDR) strains (they exhibited resistance to antibiotics from more than three antimicrobial classes). Similarly, all *E. coli* isolates from seafoods in Lagos Nigeria showed high level of resistance (85.7–96.1%) to tetracycline and trimethoprim, however, isolates were highly susceptible to ciprofloxacin, amikacin, imipenem and cefepime (Odumosu et al. 2021). In Mumbai, India, 71.58% of 475 *E. coli* isolates obtained from fresh seafood in retail markets exhibited high extended spectrum B-lactamase resistance; cefotaxime (95%), ceftazidime (90.29%), and cefpodoxime (90.88%), however, isolates showed considerable susceptibility to cefoxitin (66.76%), imipenem (74.41%), and meropenem (51.18%) (Singh et al. 2020). These findings are especially alarming due to the risk of disseminating multidrug-resistant *E. coli* strains to seafood consumer, thus improving the hygiene of coastal waters is quite essential.

Findings in the present study may suggest that shellfish could be possible spillover reservoirs of resistant and virulent STEC, and improperly heat-treated edible shellfish may be a probable source of infection for consumers in the study area. Additional future STEC surveillance and interdisciplinary coordinated efforts are essentially needed to protect coastal water and prevent STEC outbreaks associated with the human consumption of these invertebrates.

## Data Availability

All data generated or analyzed during this study are included in this published article.
